# High-accuracy measurement, advanced theory and analysis of the evolution of satellite transitions in manganese *K*α using XR-HERFD

**DOI:** 10.1107/S2052252524005165

**Published:** 2024-06-21

**Authors:** Daniel Sier, Jonathan W. Dean, Nicholas T. T. Tran, Tony Kirk, Chanh Q. Tran, J. Frederick W. Mosselmans, Sofia Diaz-Moreno, Christopher T. Chantler

**Affiliations:** a School of Physics, University of Melbourne, Melbourne, Victoria, Australia; b Department of Chemistry and Physics, La Trobe University, La Trobe, Victoria, Australia; c Diamond Light Source,Didcot United Kingdom; Australian Nuclear Science and Technology Organisation and University of Wollongong, Australia

**Keywords:** inelastic X-ray scattering, X-ray absorption fine structure, computational modelling, extended-range high-energy-resolution fluorescence detection, X-ray emission spectroscopy, manganese, satellites, *K*α spectra, many-body processes

## Abstract

Here, the novel technique of extended-range high-energy-resolution fluorescence detection (XR-HERFD) has successfully observed the *n* = 2 satellite in manganese to a high accuracy. The significance of the satellite signature presented is many hundreds of standard errors and well beyond typical discovery levels of three to six standard errors. This satellite is a sensitive indicator for all manganese-containing materials in condensed matter. The uncertainty in the measurements has been defined, which clearly observes multiple peaks and structure indicative of complex physical quantum-mechanical processes. Theoretical calculations of energy eigenvalues, shake-off probability and Auger rates are also presented, which explain the origin of the satellite from physical *n* = 2 shake-off processes. The evolution in the intensity of this satellite is measured relative to the full *K*α spectrum of manganese to investigate satellite structure, and therefore many-body processes, as a function of incident energy. Results demonstrate that the many-body reduction factor *S*_0_^2^ should not be modelled with a constant value as is currently done. This work makes a significant contribution to the challenge of understanding many-body processes and interpreting HERFD or resonant inelastic X-ray scattering spectra in a quantitative manner.

## Abstract 2

Here, the *n* = 2 satellite present for manganese-containing materials and across materials science has been successfully observed by applying the new technique of extended-range high-energy-resolution fluorescence detection (XR-HERFD), developed from high-resolution resonant inelastic X-ray scattering and HERFD, and the spectra have been predicted with new advanced theory.

## 1. Introduction

X-ray absorption spectroscopy (XAS) is a major and extremely powerful technique, and one of the mainstays of synchrotrons and beamlines, together with powder and single-crystal crystallography. Thousands of papers are published on XAS per annum in many major high-profile journals. Within XAS lies several sub-categories such as X-ray absorption fine structure (XAFS), including both X-ray absorption near-edge structure (XANES) and extended XAFS (EXAFS), each implemented in both transmission and fluorescence modes. Closely related is X-ray emission spectroscopy (XES). In recent decades, exciting beamlines with energy resolutions lower than the absorption hole width (and typically less than 1 eV) have defined resonant inelastic X-ray scattering (or spectroscopy) (RIXS) and high-energy-resolution fluorescence detection (HERFD) for high-resolution maps of the pre-edge, edge and near-edge structure (Sparks, 1974 ▸; Eisenberger *et al.*, 1976 ▸; Blume, 1985 ▸; Hämäläinen *et al.*, 1991 ▸; Carlisle *et al.*, 1995 ▸; Kao *et al.*, 1996 ▸; Glatzel & Bergmann, 2005 ▸). Great insight has been found for chemical sensitivity and dependence upon local environment bonding, oxidation state and symmetry (Kotani & Shin, 2001 ▸; Ament *et al.*, 2011 ▸; Glatzel *et al.*, 2013 ▸; Gel’mukhanov *et al.*, 2021 ▸), and for superconductivity (Lee *et al.*, 2014 ▸), charge-transfer behaviour (Bisogni *et al.*, 2016 ▸), Mott insulators (Ivashko *et al.*, 2019 ▸), catalysis (Timoshenko & Frenkel, 2019 ▸) and battery hysteresis (House *et al.*, 2020 ▸). There are persisting challenges in explaining structure beyond the position of peaks on a relative energy scale, including the valence-to-core spectroscopy and the presence of Coster–Kronig and resonant Auger transition processes (De Groot, 1996 ▸).

However, there is much more exciting scientific content in the data, which is only now beginning to be recognized and observed using extended-range HERFD (XR-HERFD). Whilst RIXS works with high resolution and fine energy steps in incident and emission energies near to and below the edge, XR-HERFD looks in places where no signature might have been seen before, for critical quantum processes that define bonding and quantum chemistry. In particular, higher-order relativistic quantum theory is necessary for much of the advanced phenomena we observe today, and we will see this need even in XAS. XR-HERFD reveals many-body processes and distinct satellites in the spectra, not before seen in XAS, XAFS, XANES or RIXS. A satellite in the context of XES data is described by a feature in the data that has an energy centroid degenerate to the main, or diagram, feature. These satellites can be well resolved, or not well resolved and instead inferred from asymmetries in the diagram profile.

In standard XAFS theory, the fine structure is modelled by the following equation, originally derived by Sayers, Stern and Lytle (Sayers *et al.*, 1971 ▸; Stern, 1974 ▸) and extended afterwards to account for some approximations used in the original expression, especially including a plane-wave approximation versus a curved photoelectron wave (Lee & Pendry, 1975 ▸; Gurman *et al.*, 1984 ▸; Binsted *et al.*, 1986 ▸), inclusion of multiple-legged paths and Green’s functions, a Gaussian bond-length distribution versus asymmetric cumulants, static and dynamic disorder, and perhaps especially including the inclusion of the many-body reduction factor (Lee & Beni, 1977 ▸; Rehr *et al.*, 1978 ▸): 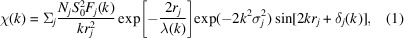
where *N*_*j*_ is the degeneracy of the path, *F*_*j*_(*k*) is the back-scattering amplitude, *r*_*j*_ = (1 + α)*r*_0*j*_ is the adjusted half-path length, (1 + α) is the thermal-expansion coefficient, *r*_0*j*_ is the input half-path length, δ_*j*_(*k*) is the phase shift and σ_*j*_ is the Debye–Waller factor, which accounts for thermal and static disorder and is defined as the mean-square variation of the scattering path length *j* [often calculated by using normal-mode eigenvectors and frequencies, though other methods such as the equation of motion can be used (Sevillano *et al.*, 1979 ▸; Poiarkova & Rehr, 1999 ▸)]. Furthermore, λ(*k*)_*j*_ is the inelastic mean free path function of the photoelectron and 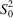, the many-body reduction factor, is assumed to be a constant.

To investigate the nature of 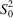 and how satellite intensity relative to the main-diagram transitions affects 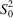, we use the XR-HERFD technique, which collects two-axis spectra over an XR of incident energies to observe novel-satellite transitions in manganese. We develop *ab initio* calculations using relativistic quantum mechanics and advanced atomic physics to investigate the origin of novel satellites for the first time.

## 2. Measurement and processing

**Figure 1:**
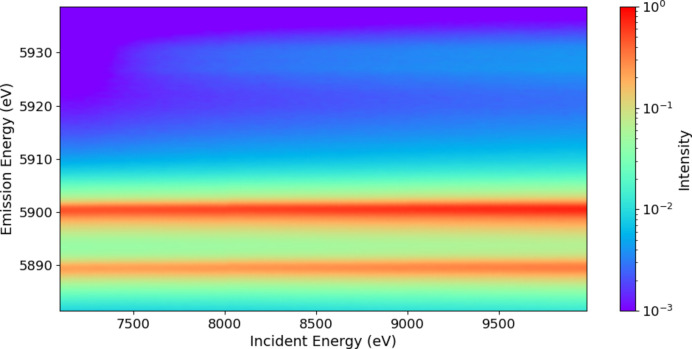
The Mn *K*α spectrum with *K*α_1_ at an emission energy *E*_em_ of ∼5900 eV, *K*α_2_ at an *E*_em_ of ∼5889 eV, and the satellite between around 5920 eV < *E*_em_ < 5935 eV. The satellite has never been observed at RIXS- or HERFD-capable beamlines, or with XAFS, because: (*a*) it does not exist at the *K* edge, but has an onset at significantly higher incident energy; (*b*) it has a small magnitude compared with the *K*α_1_ peak; and (*c*) it also occurs only in an XR emission region. Many data-collection systems lack sufficient range to find the signal and signature, and most do not obtain sufficient statistics to observe the signal. This is an argument for the value of XR-HERFD.

**Figure 2:**
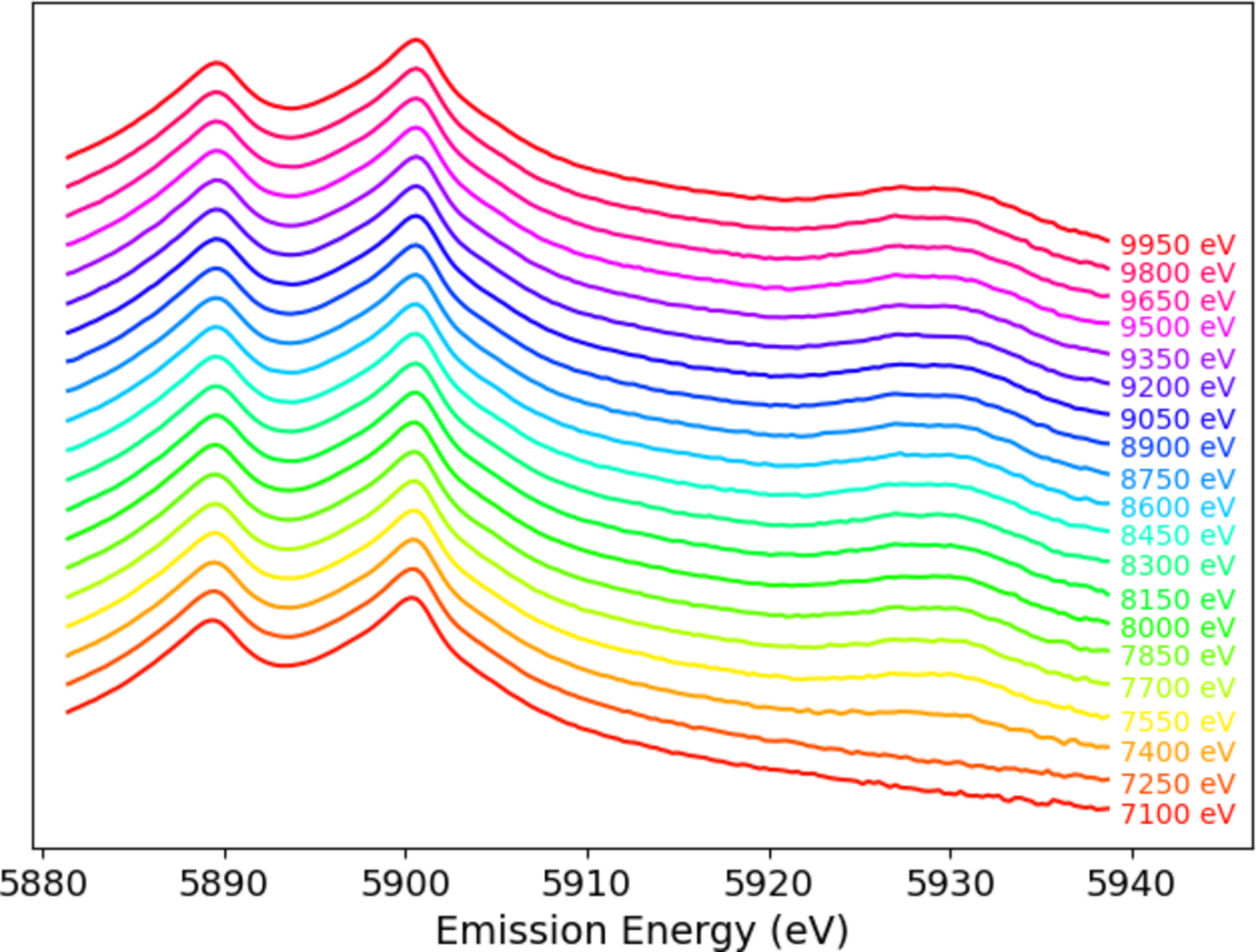
A stack plot of the XR-HERFD spectrum with labels indicating *E*_inc_, clearly revealing the onset and evolution of the satellite.

Experimental details and processing are discussed by Sier *et al.* (2024 ▸). The full raw plot of Mn *K*α for Mn metal foil shows the satellite even without processing (Fig. 1 ▸). Hence it is perhaps natural that XR-HERFD is the technique of choice to observe this type of new structure and its consequences. Contour plots hide many details that are clearly presented in a stack plot (Fig. 2 ▸), showing the onset energy for this process at an incident energy of *E*_inc_ = 7100 eV.

**Figure 3:**
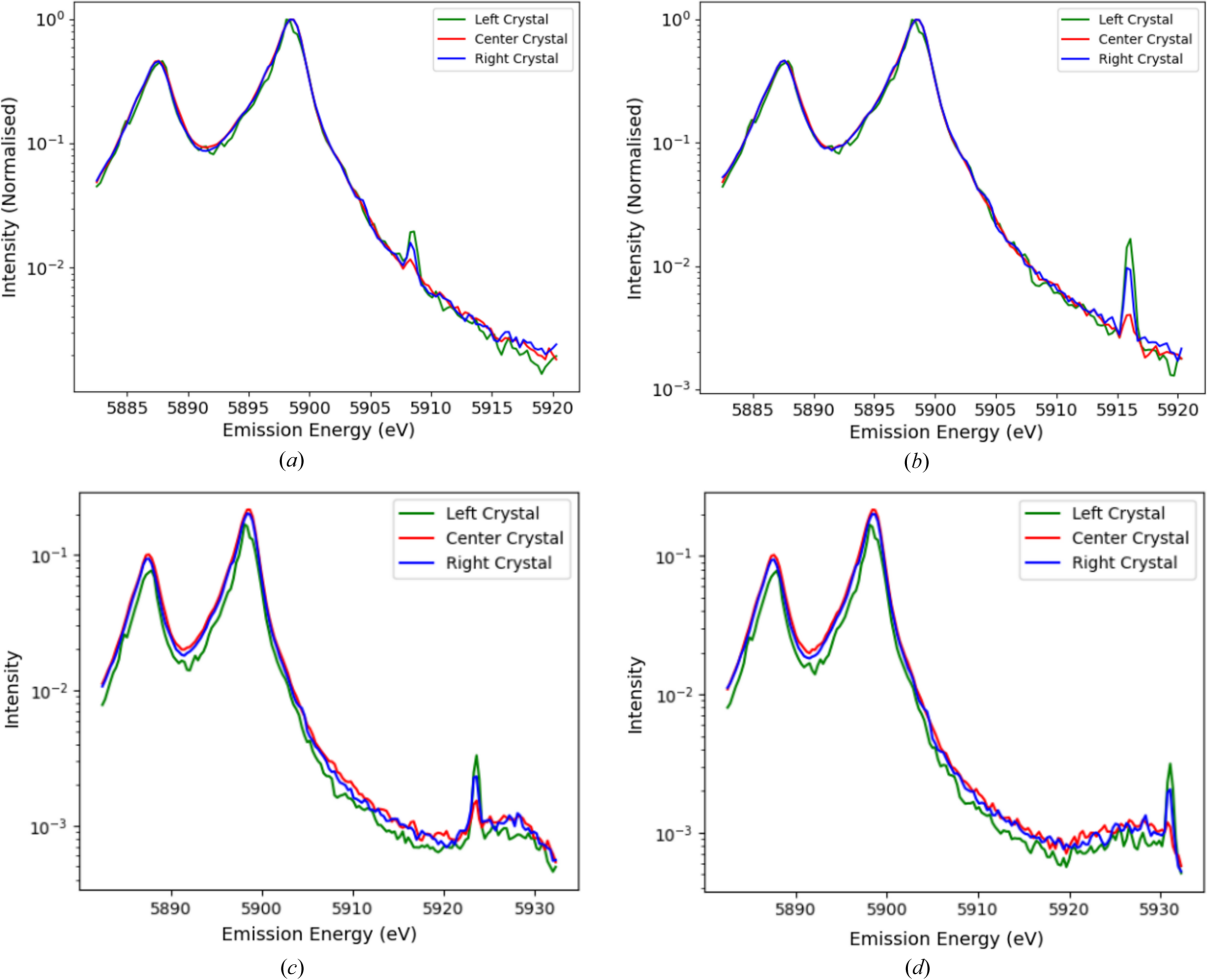
Mn *K*α spectra at incident energies *E*_inc_ of (*a*) 7880 eV, (*b*) 7890 eV, (*c*) 7900 eV and (*d*) 7910 eV, where the diffraction peaks can be observed in each of the three analyser crystals (the Bragg diffraction is from the same parallel planes normal to the crystal analyser surface but in fourth-order diffraction, and appears as a line at an angle to the fluorescence spectrum).

Data processing and analysis is crucial to understand the physics and chemistry of such processes, especially because of competing processes in low-flux regions. Fig. 3 ▸ illustrates the competition in the spectra with Bragg diffraction from elastic peak scattering, which is diffracted in fourth order rather than the third order for the main signal. This plot also displays raw-scan data quality and noise (Sier *et al.*, 2024 ▸). This is important to investigate the structure and nature of the satellite.

**Figure 4:**
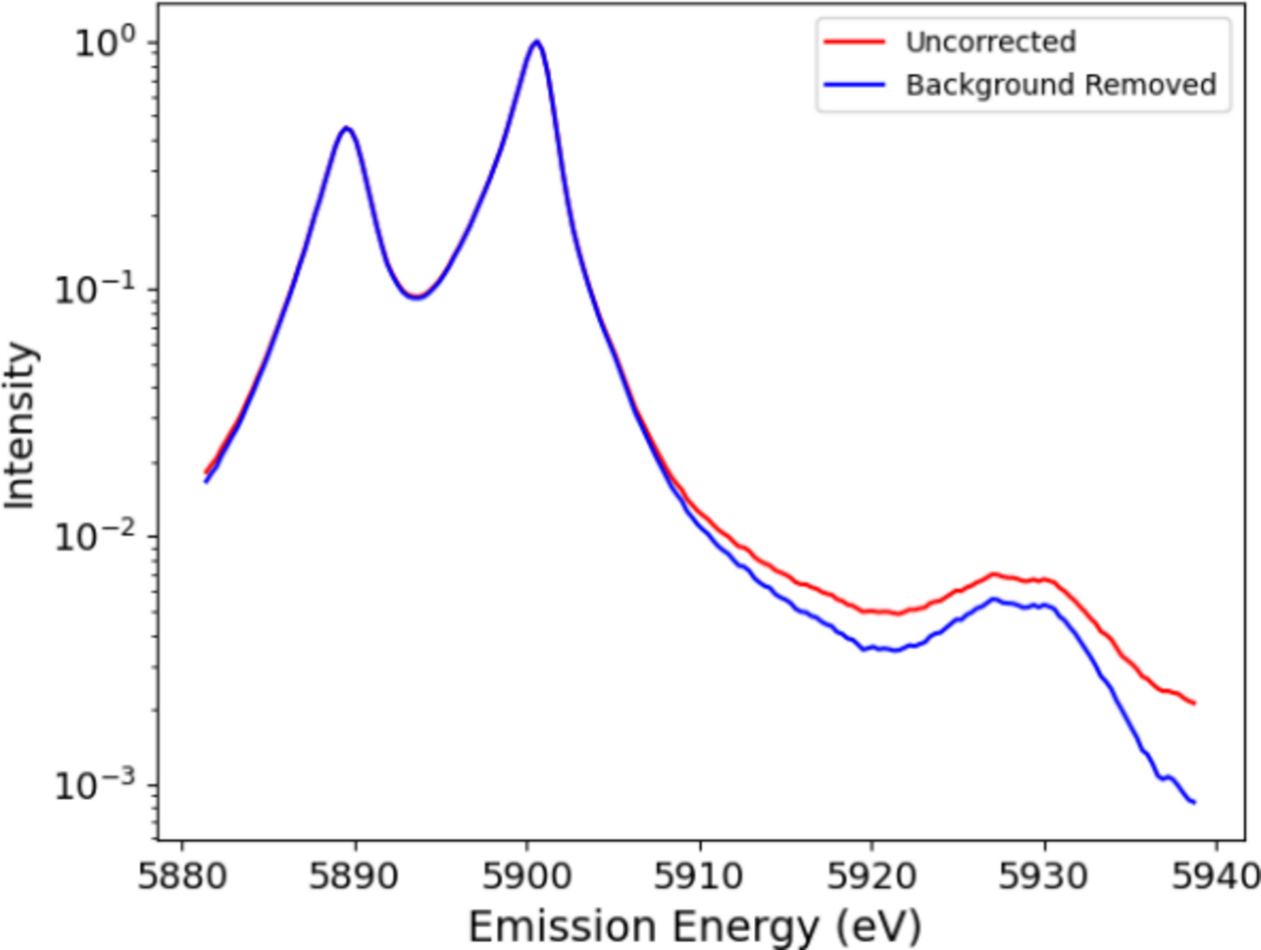
Emission spectra (XES) of Mn with (blue) and without (red) the background counts subtracted. The effect of the correction is significant in the low-intensity satellite region where the peak magnitude is reduced by 21.6%.

**Figure 5:**
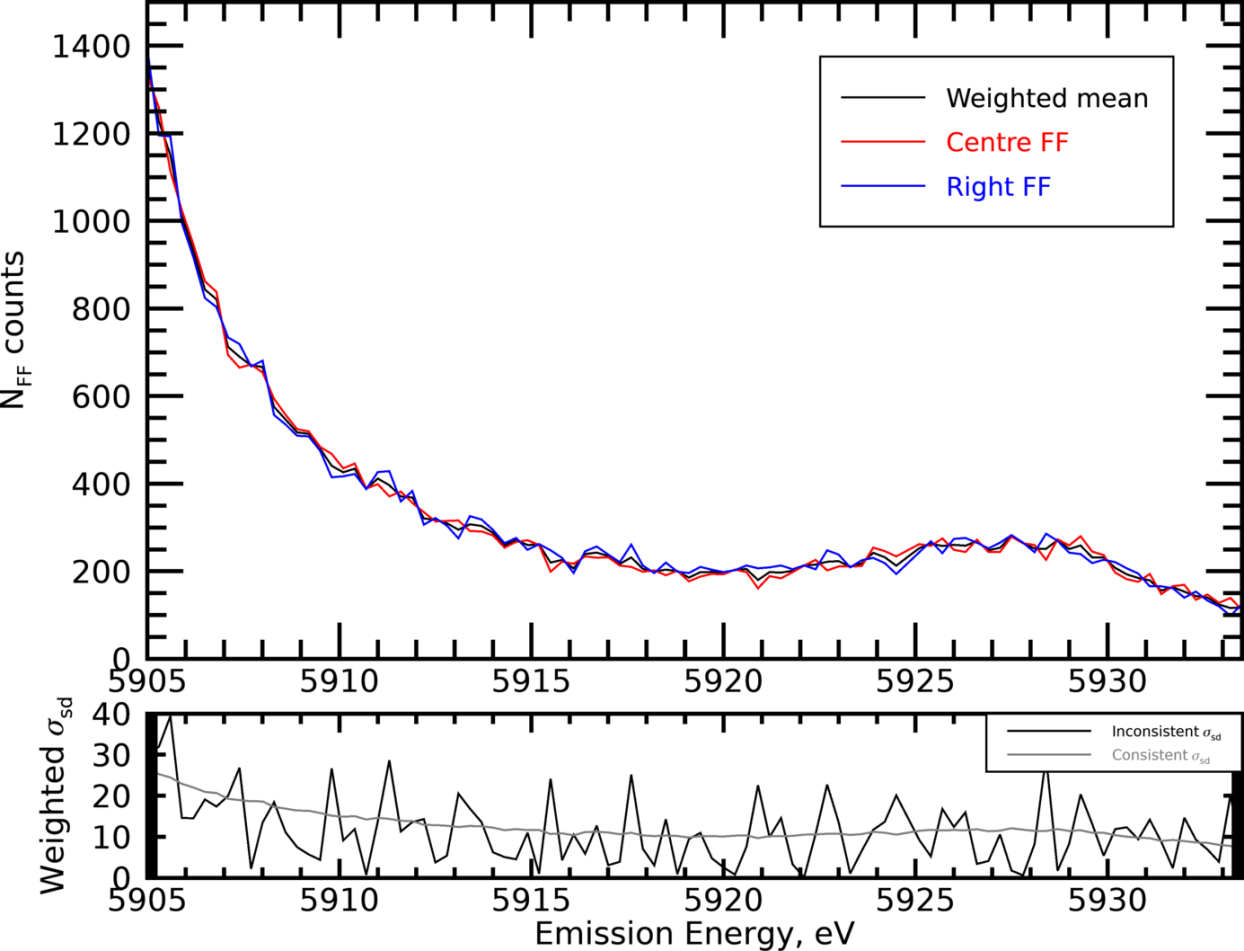
(Above) Fluorescence counts in the centre and right crystal analysers, *N*_FF_. (Below) A comparison of ‘consistent’ and ‘inconsistent’ standard deviation (variance) measures of σ_sd_ versus *E*_em_, for the centre and right crystal analysers after scaling in the tail region of the *K*α_1_ spectrum. The ‘inconsistent’ calculation oscillates around the ‘consistent’ estimate and indicates that the consistent method is a good upper bound for the uncertainty of the weighted mean.

Similarly, there is a significant background spectrum seen in the used Medipix detector in the background that has structure and an onset of its own, arising from (direct) scatter into the detector, which can be measured and isolated (Fig. 4 ▸), and can be seen to have a significant impact upon the shape and structure of the satellite. When considering any data in science, we must aim to define and present uncertainties from both statistics and systematic sources, from both noise and variance. There are two main approaches for combining datasets based upon assumptions of consistency of the datasets or inconsistency of the datasets (Sier *et al.*, 2024 ▸). Here, they are presented for the spectral region of the satellite and are shown to be highly consistent with one another (Fig. 5 ▸), which is a strong commendation for the stability of the beamline.

Crucial to this analysis was the use of HDF binary data files for processing using the full images and spectra, and the separation of image locations for the individual analyser crystals of the HERFD analyser on the detector. Armed, then, with well defined uncertainty including precision and accuracy, we can investigate the structure experimentally and theoretically. Importantly, we can isolate the Mn *K*α_1,2_ spectrum without any assumptions about the shape or structure of the main spectrum. That is, we can isolate it using XR-HERFD according to the experimental spectra directly with neither theoretical input nor assumption.

### 2.1. Isolation of satellite

**Figure 6:**
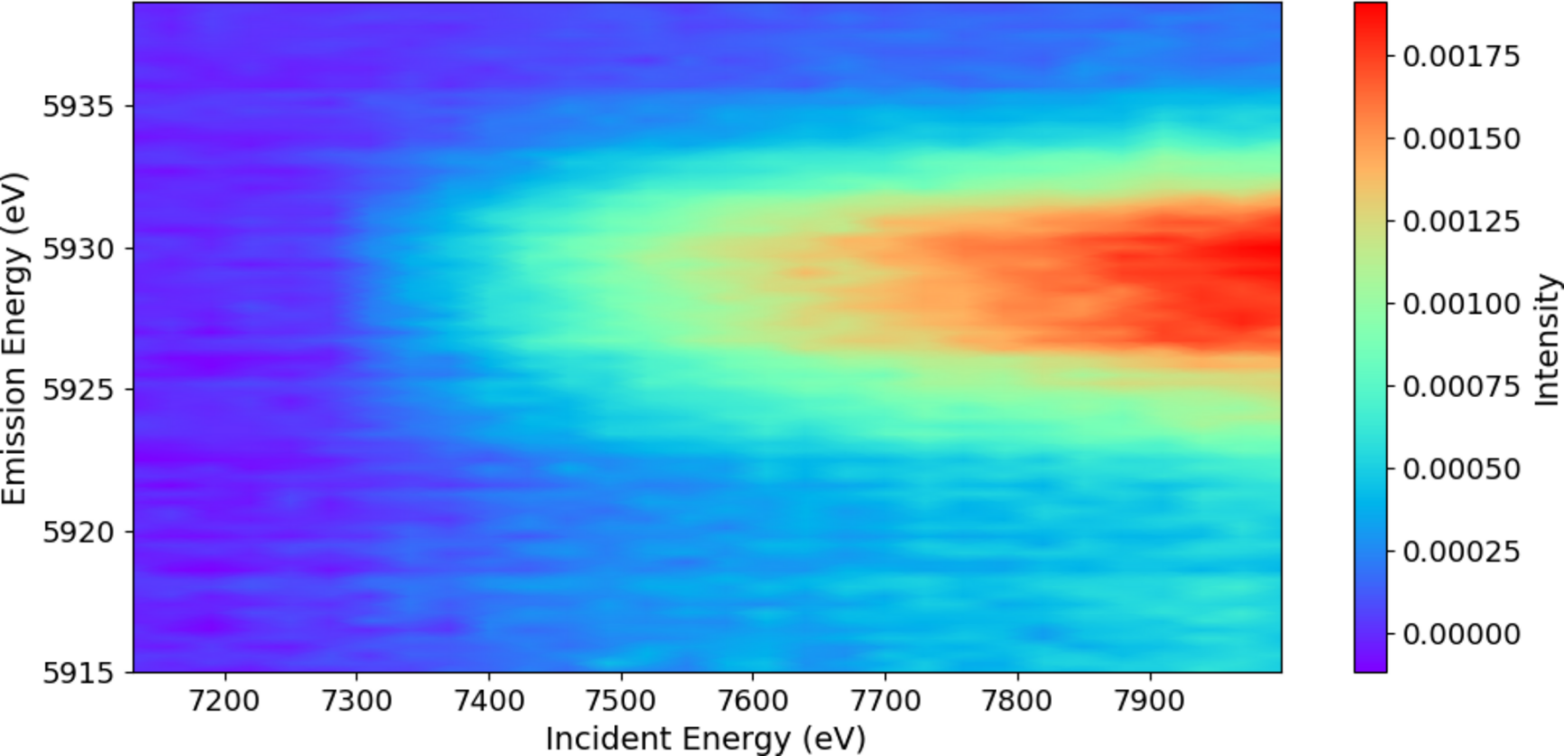
The *n* = 2 satellite observed, isolated from the Mn *K*α_1_ background, showing the increase in intensity with energy above the onset.

**Figure 7:**
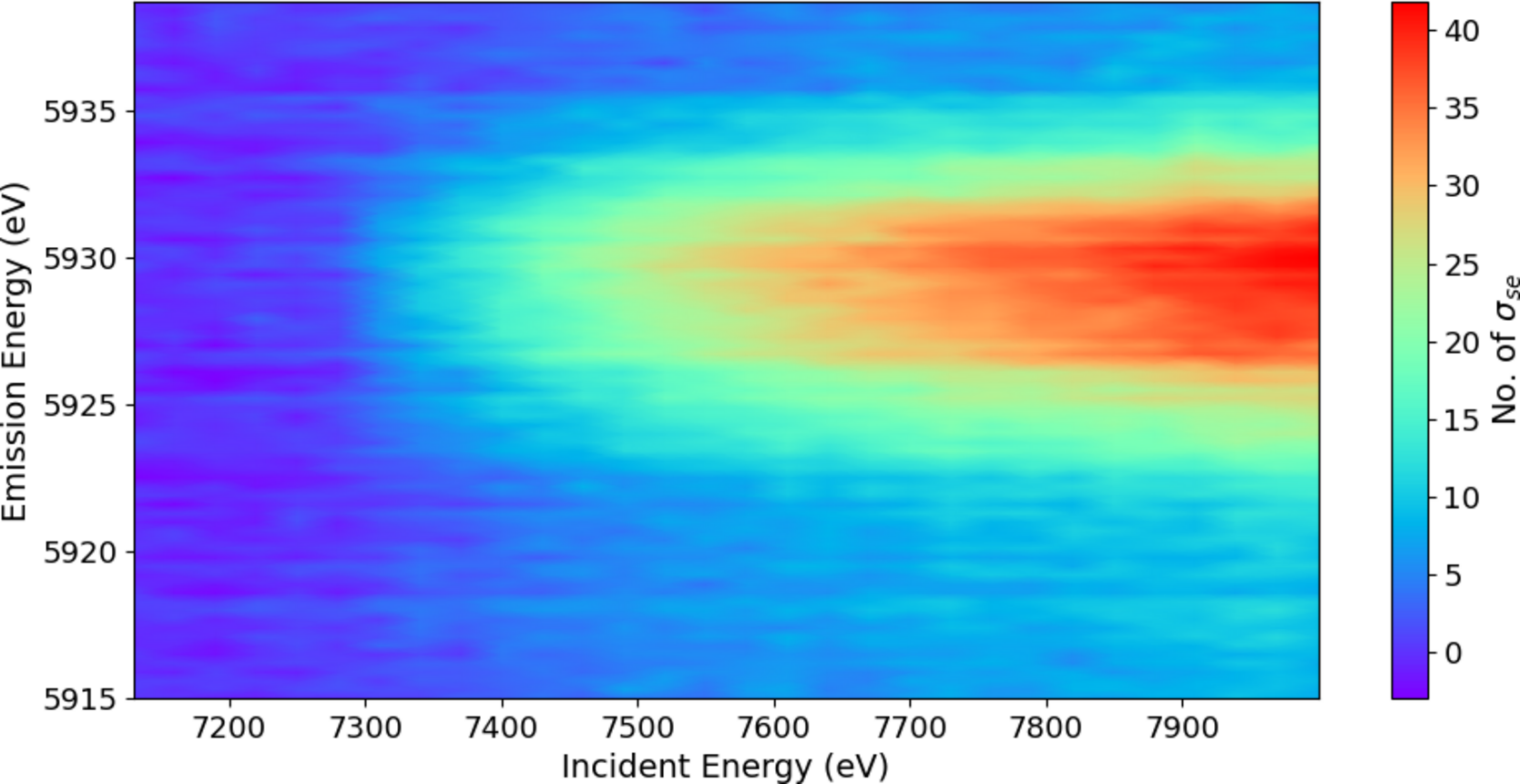
A significance plot of the signature of the satellite divided by the standard error uncertainty of the pooled data. The colour legend in number of standard errors σ_se_ gives the significance of each data point.

By subtracting the main *K*α background, we can reveal the novel-satellite spectrum (Fig. 6 ▸) with the corresponding map of the significance of the spectrum using the standard error uncertainty arising from the consistent pooled data of the crystal analysers (Fig. 7 ▸). Appendix *A*[App appa] discusses the improved statistics and significance of the current experiment.

### 2.2. Isolated satellite with explicit structure and significance

**Figure 8:**
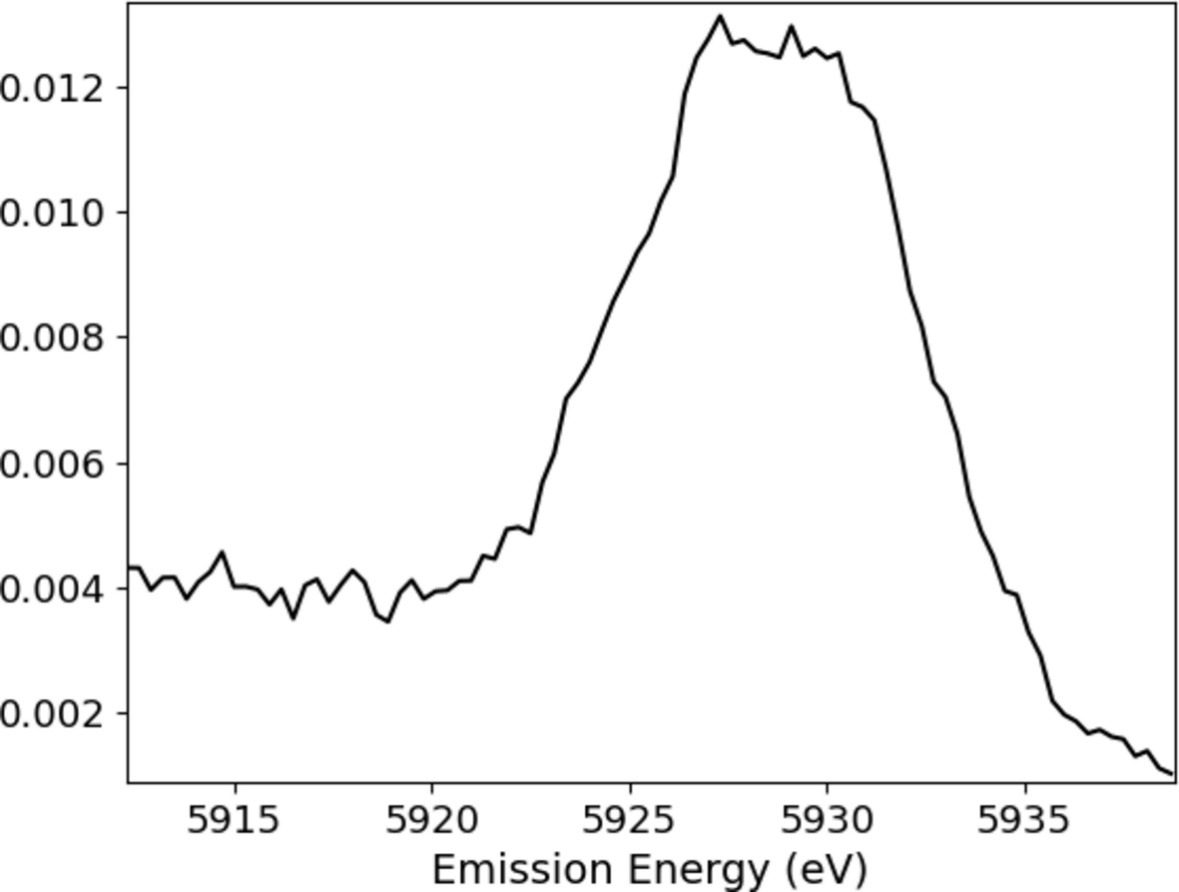
The *n* = 2 satellite with experimental Mn *K*α_1,2_ background subtraction to show the satellite structure and the implication of a third-peak region, using the highest three experimental energies in the range 9800–10 000 eV.

**Figure 9:**
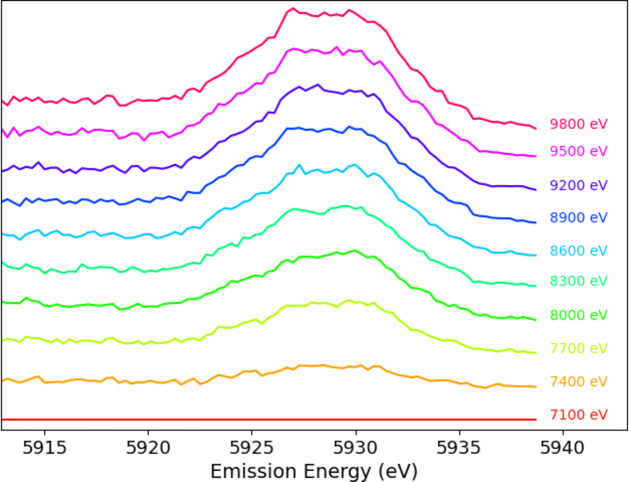
The *n* = 2 satellite with experimental Mn *K*α_1,2_ background subtraction to show the satellite structure and the implication of a third-peak region, with evolution with incident energy. Each XES spectra is vertically offset by a constant amount for clarity.

When the experimental background subtraction is performed, the main ‘double peak’ is very clear, and the statistics can be improved by pooling *e.g.* three incident energies in the range 9800–10 000 eV (Fig. 8 ▸). However, this also clearly indicates a third weaker peak region at lower energy. Fig. 9 ▸ demonstrates that this region increases at the same rate as the double-peak structure, and hence may have the same onset and cause. The significance of this third feature is indeed limited by the statistics, and for an individual incident energy is a small number of standard errors above the background. There are experimental limitations of the subtraction of *K*α_1,2_ and this can be investigated further. However, the experimental data give very strong information and structure suitable for advanced theoretical inquiry.

## 3. Origins of the satellite

Atomic emission spectrometry has investigated characteristic X-ray spectra for over a century. In these experiments, the incident energy is rarely a topic of discussion, with the incident photon just assumed to be far above the *K*-edge energy, typically at least three times. The satellite spectrum with energy slightly above the *K*α_1_ peak is historically labelled as the *K*α_3,4_ satellite spectrum, where the label simply indicates that they were the third and fourth to be identified (Siegbahn notation).

The origin of this *K*α_3,4_ satellite has had many hypotheses: higher-order electron transitions, such as electric quadrupole (E2) and magnetic dipole (M1) transitions; solid-state effects; Kondo-like transitions; impurities in the samples; and secondary ionization. Secondary ionization is sometimes referred to as shake off as it occurs when a second electron is ‘shaken off’ into the continuum. Early observations of a *K*α_3,4_ satellite (Wentzel, 1921 ▸) yielded early attribution of the satellite to originate from an *n* = 2 secondary ionization (Kennard & Ramberg, 1934 ▸). In titanium, the satellite would appear, and its intensity would increase shortly after the incident energy was greater than the 2*s* and 2*p* binding energies in addition to the *K*-edge energy (Parratt, 1936 ▸). The shape and intensity of the satellite have only been investigated recently, and only for copper (Deutsch *et al.*, 1996 ▸; Melia *et al.*, 2023 ▸).

If the *K*α_3,4_ satellite is the product of a double ionization event – that is, if it is a many-body process – then there are direct consequences for the 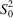 parameter in the standard XAFS equation [equation (1)] and how to use it. Therefore, this is a major area of potential inquiry that lies at the intersection of state-of-the-art relativistic atomic physics, condensed matter physics, synchrotron science and molecular science.

For many decades it has been recognized that if this spectrum relates to an *n* = 2 satellite then logically there should be 2*s*_1/2_, 2*p*_1/2_ and 2*p*_3/2_ components. However, experimental and theoretical evidence for all these decades have shown that the spectrum is purely a 2*p* satellite spectrum (Deutsch *et al.*, 1996 ▸). There has been no experimental or theoretical investigation of manganese, so these claims have been based only on copper, *Z* = 29. If only 2*p*, then where is the 2*s* spectrum and why? Hence, if either and both spectra can be observed, a key question concerning the *K*α_3,4_ satellite and its origins is the ratio of the 2*s* or 2*p* shake-off events.

### 3.1. *Ab initio* transition calculations

To determine the origins of the observed physical process, high-accuracy calculations are performed and fitted to the experimental data. The eigenvalue spectrum for the 2*s* and 2*p* shake-off satellites and their relative amplitudes are calculated using the multiconfiguration Dirac–Hartree–Fock (MCDHF) method. The MCDHF method is implemented through the general relativistic atomic structure software package *GRASP* (Chantler *et al.*, 2014 ▸; Froese Fischer *et al.*, 2019 ▸; Jönsson *et al.*, 2023*a* ▸,*b* ▸). This approach is fully relativistic with *jj* coupling core wavefunctions using the Lowe–Chantler–Grant (LCG) self-energy (Nguyen *et al.*, 2023 ▸).

**Figure 10:**
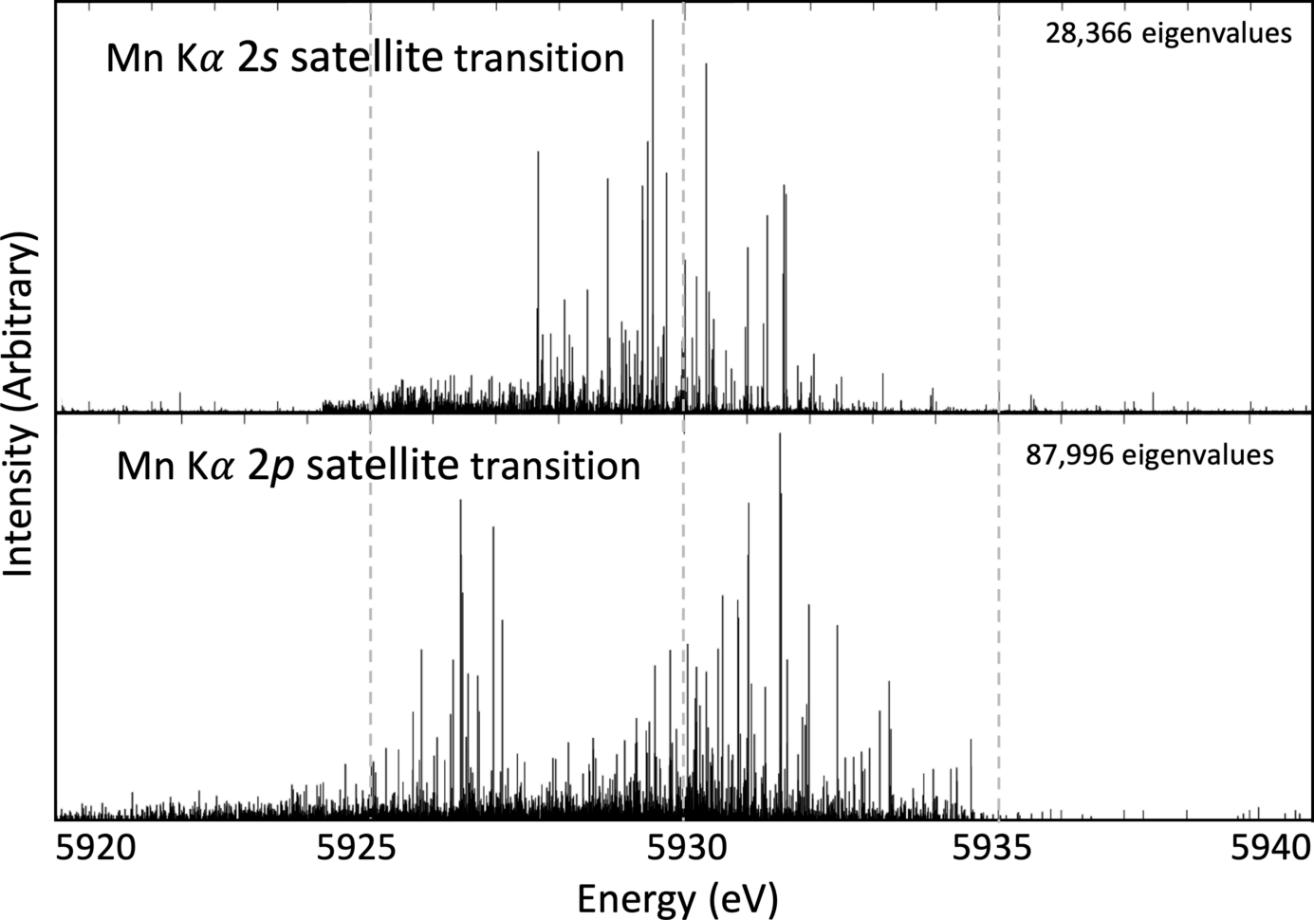
The eigenvalue emission spectra (XES) for the Mn *K*α 2*s* and 2*p* shake-off transitions. These are hypothesized to be the origin of the *K*α_3,4_ satellite. There is not one peak for either of the 2*s* or 2*p* satellite spectra; rather, there are 28 366 discrete energy eigenvalues for the 2*s* satellite transition and 87 966 eigenvalues for the 2*p* satellite transition. The height represents the relative probability of each eigenvalue within the transition. Each energy eigenvalue experimentally yields a broadened (Lorentzian) profile with area corresponding to the amplitude. This figure details the current best and first theoretical prediction of the complex spectrum observed.

Manganese has a complex canonical ground-state electron configuration of [Ar]3*d*^5^4*s*^2^, which has the maximally allowed number of unpaired electrons in the 3*d* orbital, rendering all such theoretical computations extremely challenging by any approach. For a *K*α transition, the MCDHF approach calculates the atomic wavefunction for the initial [1*s*] state and final [2*p*] state, where square brackets denote holes. The *K*α_3,4_ profile, as the result of *n* = 2 shake-off satellite transitions, is therefore modelled with the transitions [1*s*2*s*] → [2*p*2*s*] for the 2*s* shake-off transition and [1*s*2*p*] → [2*p*^2^] for the 2*p* shake-off transition. Once initial and final states are calculated, energy eigenvalues and relative intensities are obtained through biorthogonalization. Recent work outlines the success of this approach for scandium (Dean *et al.*, 2022 ▸) and copper (Nguyen *et al.*, 2022*a* ▸,*b* ▸). Results for the Mn *K*α 2*s* and 2*p* shake-off satellites are presented in Fig. 10 ▸.

Due to the complex open-shell structure of atomic manganese, there are many ways to couple the electron spin, yielding many different transition energies. The eigenvalue spectra in Fig. 10 ▸ contain tens of thousands of independent eigenvalues. Each eigenvalue represents a different spin coupling resulting in non-degenerate transition energies. We represent the probability of each eigenvalue by the relative height of the eigenvalue within the transition.

Each eigenvalue is convolved with a Lorentzian profile where the energy of the eigenvalue is the Lorentzian centroid *E*_*n*_ and with an amplitude or integrated area *b*_*n*_, given by the relative height of the eigenvalue peak. Since this work fits two transition spectra, there is a further *t* subscript to denote which transition the *N*th eigenvalue and amplitude belongs to, *E*_*n*,*t*_ and *b*_*n*,*t*_, where *t* ∈ {2*s*, 2*p*}. The full width at half-maximum, γ_*t*_, is left as a free parameter, consistent for each eigenvalue within the same transition. The full profile is therefore 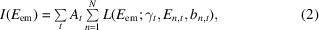
where *A*_*t*_ is the relative amplitude of the transition, either 2*s* or 2*p* in this work. This amplitude parameter, *A*_*t*_, is calculated *ab initio* following Melia *et al.* (2023 ▸), using the wavefunctions of the initial and final states to calculate the probability of a 2*s* or 2*p* shake-off event.

Here, we investigate five different hypotheses for the Mn *K*α_3,4_ satellite: (1) the spectrum is a 2*s* shake-off satellite only, (2) the spectrum is a 2*p* shake-off satellite only (following past literature for copper), (3) the spectrum is composed of both shake-off satellites with *A*_*t*_ given by the theoretical *ab initio* shake probability, (4) an investigation of other decay processes or simply (5) the spectrum is composed of both shake-off satellites with *A*_*t*_ as an arbitrary free parameter. These five models were fitted against the *K*α_3,4_ spectrum for each incident energy, and the goodness-of-fit 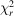 measure is presented in Fig. 13. The 2*s* shake-off satellite alone (red), hypothesis (1), cannot fit the satellite. The 2*p* shake-off satellite (orange), hypothesis (2), represents the main two peaks but not the shoulder or the profile shape. However, the best fits are when both 2*s* and 2*p* satellites are included using the theoretical *ab initio* shake probability.

**Figure 11:**
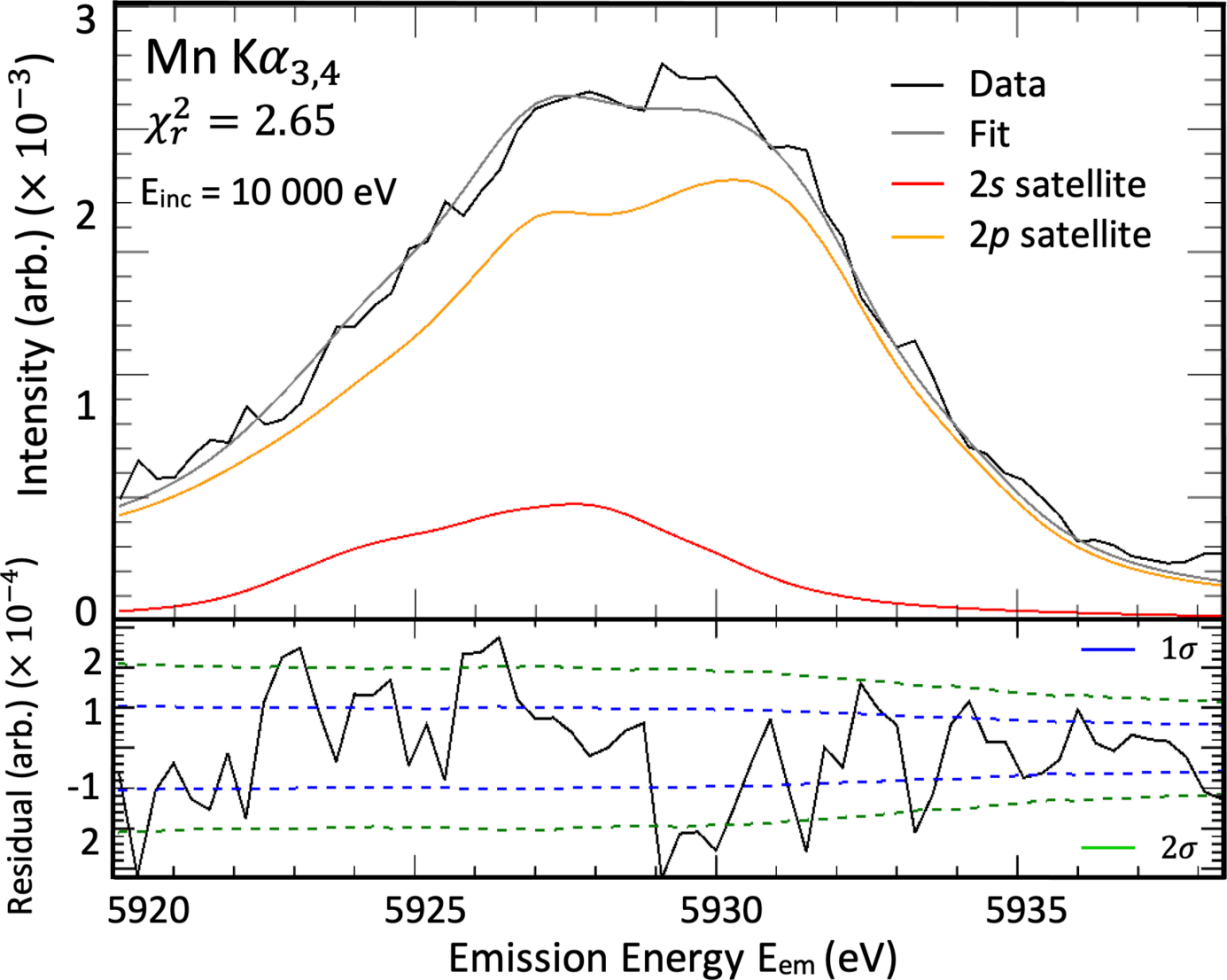
A fit of the satellite XES (*E*_em_) using the *ab initio* shake-off values as the relative intensities, *A*_*t*_ in equation (2). The background spectrum has been subtracted so we only have the *K*α_3,4_ profile with no diagram spectrum. The incident energy is *E*_inc_ = 9100 eV. The 2*s* spectrum has quite the wrong structure and would require a significant energy offset and correction. The 2*p* theoretical spectrum predicts the two main peaks with accurate energies, but predicts a different peak shape from experiment and omits the shoulder. Including both components and using the theoretical *ab initio* shake probabilities for their relative amplitudes represents the experimental data well, 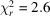.

Here, the *ab initio* shake-off probabilities are 0.168% for a 2*s* shake off and 0.940% for a 2*p* shake off. These values are quite different from past predictions: Mukoyama & Taniguchi (1987 ▸) predicted 0.134% for 2*s* and 0.669% for 2*p*,while Kochur *et al.* (2002 ▸) predicted 0.26% for 2*s* and 1.17% for 2*p*. We discuss these predictions in a separate paper. Since this work considers the background-subtracted *K*α_3,4_ spectrum, we normalize the probabilities such that the sum of probabilities for a 2*s* and 2*p* shake off is unity. The probability of a 2*s* shake off is 15.16% and the probability of a 2*p* shake off is 84.84%. Fig. 11 ▸ shows the fit using these values as the relative intensities in equation (2): *A*_2*s*_ = 0.1516 and *A*_2*p*_ = 0.8484.

### 3.2. *Ab initio* non-radiative processes

A previous study of the *K*α_3,4_ spectrum in copper found no 2*s* shake-off satellite intensity (Deutsch *et al.*, 1996 ▸). This is opposed to theoretical calculations modelled by the shake-off probability, which is calculated with the adiabatic, or sudden, approximation (Mukoyama & Taniguchi, 1987 ▸), suggesting a value closer to 25% of the *K*α_3,4_ spectrum. Recent work has suggested that this latter prediction neglects other decay mechanisms (Melia *et al.*, 2023 ▸). A shake-off satellite photon is only observed if the 2*p* → 1*s* electron transition takes place before the satellite vacancy is filled. A common process for filling an electron vacancy is the non-radiative Auger process. Accounting for the Auger decay channels was critical to investigate the controversy between theoretical and experimental satellite intensities for the 2*s* shake-off satellite in copper (Melia *et al.*, 2023 ▸). In this work, we now investigate this hypothesis by calculating the Auger suppression factor for manganese 2*s* and 2*p* shake-off satellites.

**Figure 12:**
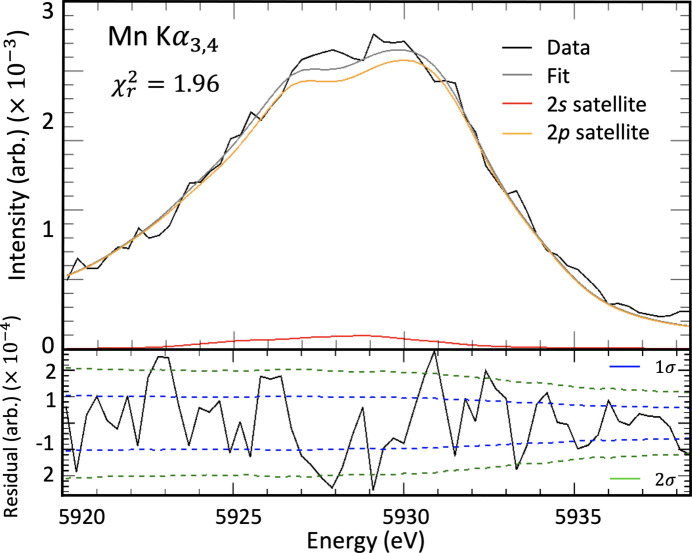
Fitting the experimental XES (*E*_em_) using the theoretically derived spectrum for the 2*s* and 2*p* shake-off satellites to the *K*α_3,4_ experimental profile for the maximum incident energy *E*_inc_ = 9100 eV. Auger processes have been considered, and the difference between this figure and Fig. 11 ▸ is the relative intensities of the 2*s* and 2*p* shake-off satellites. This improved 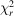 indicates that the Auger process is important. This shows that the *K*α_3,4_ XES spectrum is produced by ionization of the 2*p* satellite, indeed with a contribution from 2*s* ionization, along with the 1*s* electron. Hence, this profile is well predicted as the result of many-body processes involving double ionization.

To calculate the Auger suppression factor, the non-radiative rates for the two different initial states, [1*s*2*s*] and [1*s*2*p*], must be considered. *RATIP* software (Fritzsche, 2012 ▸) calculates the rates in conjunction with *GRASP*. A [1*s*2*s*] excited state has a radiative decay rate of 0.119 eV ℏ^−1^ and a total nonradiative Auger rate of 14.014 eV ℏ^−1^. For the [1*s*2*p*] excited state, the radiative decay rate is 0.257 eV ℏ^−1^ and the nonradiative rate is 5.299 eV ℏ^−1^. This leads to a 2*s* Auger suppression factor of 0.119/14.014 = 0.0085 and a 2*p* Auger suppression factor of 0.257/5.299 = 0.0485. The shake-off probabilities are multiplied by the Auger suppression factor and then renormalized. This results in the expected satellite spectrum intensity, *A*_*t*_, which will be used in equation (2). The values obtained are *A*_2*s*_ = 0.0304 and *A*_2*p*_ = 0.9696. The reduction in the intensity of the 2*s* satellite compared with the 2*p* satellite is understood – the [1*s*2*s*] double-hole excited state is significantly more likely to relax via emission of an Auger electron than a satellite photon, roughly 120 times as likely. Compare this with the [1*s*2*p*] excited state where relaxation via an Auger electron is only of the order of 20 times more likely than the radiative photon pathway. Using the *ab initio* shake-off probabilities results in a 2*s* shake-off intensity prediction far greater than that observed experimentally. Fig. 12 ▸ shows the results when fitting the Auger processes, satellite intensities significantly improved compared with omitting the Auger suppression. The fully free fit, while less physical, does not have a significantly improved 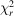, indicating that the dominant physical processes have been correctly represented by theory in Fig. 12 ▸.

**Figure 13:**
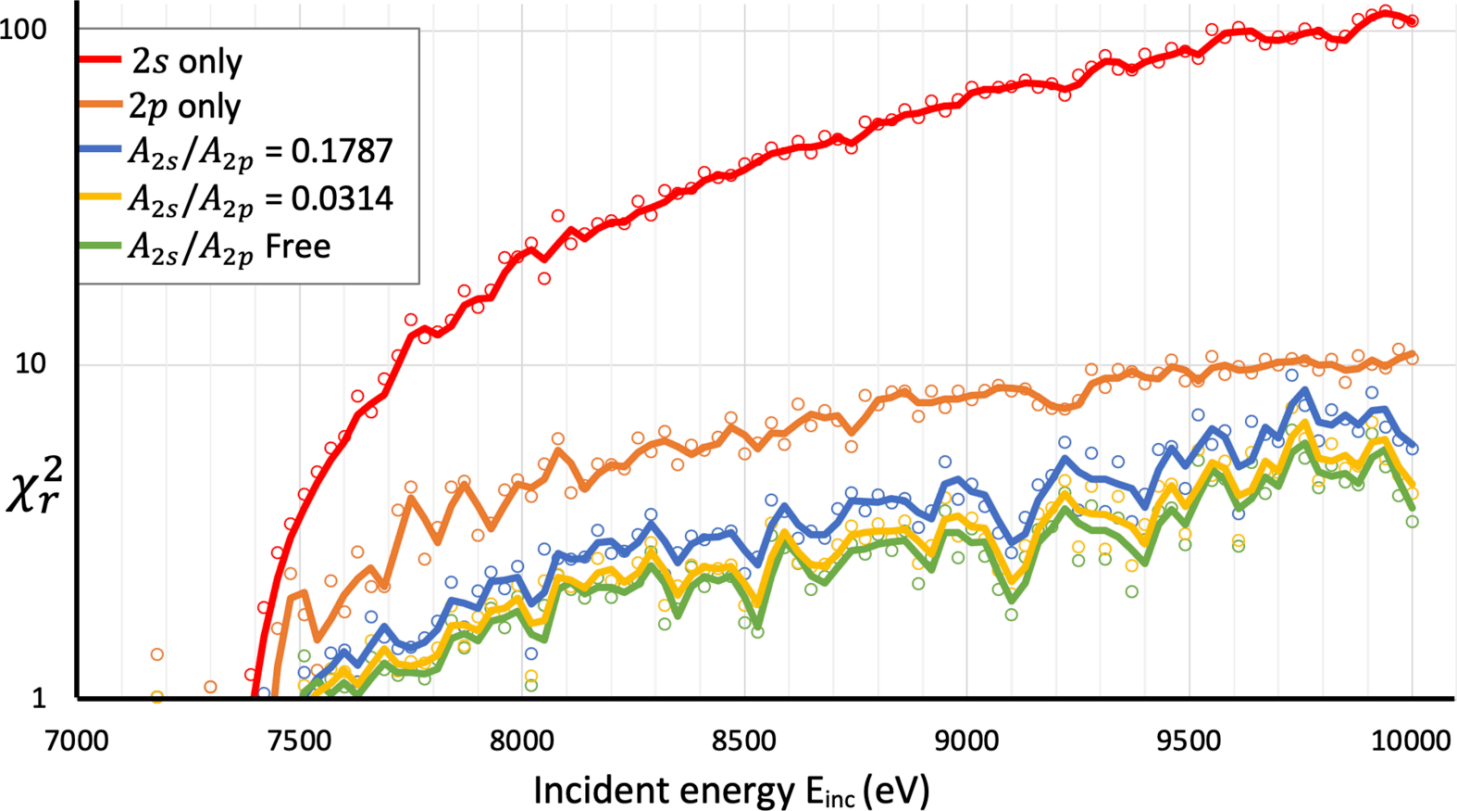
A log plot of 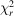 for each of the five models for each incident energy. The five models fitted to the *K*α_3,4_ spectrum are: the 2*s* shake-off satellite alone; the 2*p* shake-off satellite alone; both satellites with intensity ratio *A*_2*s*_/*A*_2*p*_ = 0.1787, fixed by shake-off prediction; both satellites with *A*_2*s*_/*A*_2*p*_ = 0.0314, using our shake-off prediction with Auger suppression; and both satellites with *A*_2*s*_/*A*_2*p*_ as a free parameter. Coloured lines represent the two-point moving average of the adjacent points of corresponding colour. The fit is very poor for the 2*s* satellite alone but is improved upon for the 2*p* satellite alone; however, the fits with both are better. The fits for the Auger corrected intensity ratio are a significant improvement on the non-corrected model. The free-parameter model fit is not a significant improvement on the Auger corrected model. The value for the *A*_2*s*_/*A*_2*p*_ ratio as a free parameter is shown in Fig. 14 ▸.

However, using the five models, we can investigate the spectral components as a function of energy. We use each *K*α_3,4_ spectrum from incident energy 8830 eV to 10 000 eV, spaced 30 eV apart (40 different *K*α_3,4_ spectra). In the sudden or impact limit at high energy, they should agree with theoretical shake predictions, as indeed they do. However, the general improvement is valid throughout the energy range, even from the onset (Fig. 13 ▸).

**Figure 14:**
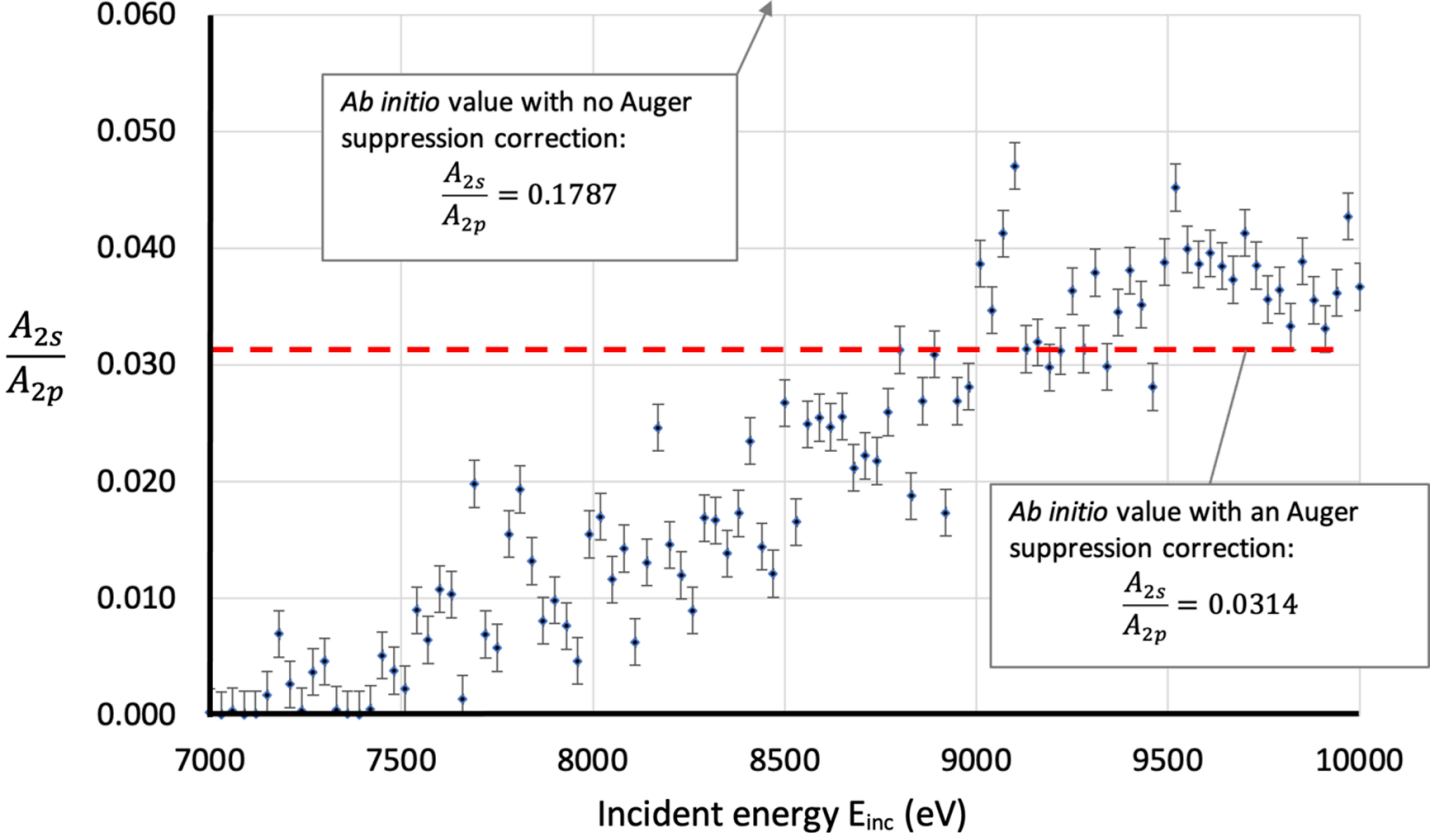
The results of the *A*_2*s*_/*A*_2*p*_ ratio when fitting the satellite intensities as a free parameter with a starting guess of 0.1787, the non-Auger suppression corrected *ab initio* value. Before the Auger suppression correction, the value is *A*_2*s*_/*A*_2*p*_ = 0.1787, more than three times higher than the chart, after the correction the value is *A*_2*s*_/*A*_2*p*_ = 0.0314, noted by the red dashed line. This is strong support for the Auger suppression factor being a real variable that must be accounted for when performing *ab initio* satellite intensity calculations.

Fig. 14 ▸ presents the *A*_2*s*_/*A*_2*p*_ ratio of these of the free fits across the function of energy. Neglecting the Auger processes yields *A*_2*s*_/*A*_2*p*_ = 0.1787; with Auger processes included, the ratio is *A*_2*s*_/*A*_2*p*_ = 0.0314. The results strongly support the significance of Auger processes in the *K*α_3,4_ spectrum.

## Controversy of 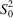


4.

Lee & Beni (1977 ▸) raised the need to consider many-body processes in XAFS analysis. They used Meldner & Perez (1971 ▸) to interpret a value of 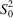 of 0.74 for neon (gas) with 6% shake-up below the edge and 20% shake-off processes to the continuum, and *e.g.* 0.43 for GeCl_4_. Carlson & Krause (1965 ▸) made theoretical estimates that suggested that the shake-off probability initially increases with energy and saturates above 150 eV above the relevant edge. They quote that these other processes will also exhibit XAFS, but the onset energy will be offset by the excitation energy of *ca* 10–30 eV. They claim that the shake-off peak is too broad to be measured. In any solid, most ‘shake-up’ processes will be due to collective plasmon excitations. Lee & Beni (1977 ▸) cite Schmidt *et al.* (1976 ▸) for an early review, primarily addressing noble gases.

This single-body versus total many-body probability ratio was defined by 1977 as 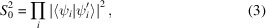
where the unprimed wavefunctions relate to the unperturbed atom and the primed wavefunctions relate to the atom with a core hole(s) present (Lee & Beni, 1977 ▸; Rehr *et al.*, 1978 ▸).

Rehr *et al.* (1978 ▸) estimated an 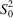 many-body reduction factor for molecules F_2_, Cl_2_ and Br_2_ as 0.60 ± 0.04, 0.64 ± 0.04 and 0.64 ± 0.04, respectively. These were substantially different from their corresponding atomic computations for free F, Cl and Br atoms of 0.74 ± 0.04, 0.71 ± 0.04 and 0.72 ± 0.04, respectively. They suggested that these values might be applicable in the high-energy ‘sudden’ or ‘impact’ limit, suggesting that above about 200 eV above the edge the EXAFS should be reduced for many-body effects by a constant factor of 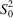, which would not apply near the XANES region. This approximation implicitly ignored XAFS from multielectron processes with probability 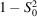. They comment that each many-body ‘channel’ should contribute an XAFS spectrum but with its own 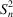 such that they sum to unity. This early computation suggests a variation of 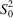 with excitation energy from 0.66 ± 0.04 to 0.74 ± 0.04 across most of the XAFS range.

Lee *et al.* (1981 ▸) discussed corrections and variations of 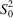 from 0.62 to 0.79, in other words much smaller than we would now currently interpret. They comment that these channels may be coherent with the single-body term so that amplitudes should add rather than probabilities. Stern (1988 ▸) provides many details on XAFS theoretical background, and tabulates 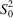 from selected atoms from He (0.73) to Sc (0.62), Fe (0.69) and U (0.73). Stern’s estimates found a strong dependence of 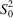 below *k* = 6, lowering from 1.00 at *k* = 6 to 0.79 ± 0.03 above *k* = 8, perhaps justifying both the difficulty of fitting low *k* and the possibility of a near-constant 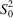. Surprisingly, Rehr *et al.* (1991 ▸) concluded that a combination of intrinsic and extrinsic losses could be combined into 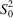 with a typical value of 0.9 (to within ±20%), with examples tabulated for GeCl_4_, Cu and Pt of 1.08, 0.85 and 0.89, respectively, mainly found by empirical fitting. More recently, Rehr & Albers (2000 ▸) stated ‘Although 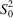 is weakly energy dependent, it is usually approximated by a constant. A fully quantum theory has yet to be developed.’ They separate ‘extrinsic losses’ reflected in the path-dependent inelastic mean free path and often dominated by plasmons, and recommend that they be defined within a complex energy-dependent ‘self-energy’ 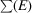 to give a real energy shift and a decay. Then the ‘intrinsic’ losses are once again represented by a constant 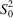. Even more recently, Fornasini (2015 ▸), Schnohr & Ridgway (2015 ▸) and Chantler & Creagh (2022 ▸) confirmed the widespread use of a constant 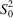. Hence, 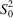, the many-body reduction factor, is assumed to be a constant. Common beamline advice in analysis and processing is that 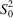 must be less than unity, and should be above *e.g.* an arbitrary 0.8. This is in the context of a constant empirical fitting factor, and with little agreement with the theoretical considerations.

When the incident energy is just above the *K* edge, only one possible ψ′ is available – the [1*s*] state, where square brackets denote a hole present in the orbital relative to the ground state. As incident energy increases above the sum of both the *K*-edge and binding energy of some *nl* electron, the probability of ejecting, shaking off, a secondary *nl* electron along with the core electron becomes non-zero. Theoretically, therefore, once shake-off processes are permitted, the probability and available ψ′ states must increase. This quantity must change with incident energy as more ψ′ possibilities become available. More generally, shake-off processes represent competing processes in the photo absorption signal, which do not show the oscillatory interference wave, and certainly not in coherent synchronization with the primary single-electron (diagram) process, thus dampening the signal by 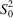.

Lee & Beni (1977 ▸) noted that measuring any change of 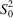 experimentally is incredibly difficult and obtaining any accurate theoretical model is also highly problematic. Therefore, the many-body reduction factor, 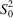 from equation (1), should be modelled as a function of incident energy, confirming quite significant variation even far above some low-*k* limit. In the current work, the nature of XR-HERFD allows the evaluation of the intensity evolution of the satellite relative to the full spectrum as a function of incident energy.

## 5. Evolution of the satellite

Equation (2) represents the theoretical XES profile for a single incident energy, which we fit to data for a single incident energy. The addition of the scaling parameter as a function of incident energy, *B*(*E*_inc_), allows for a comparison to be made with the experimental XES spectra, and between spectra of different incident energies. The equation 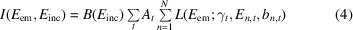
represents the two-dimensional XR-HERFD map with both XES fluorescence energy, *E*_em_, and incident energy, *E*_inc_, as variables. By fitting the XES profile for each incident energy following Fig. 12 ▸, values for *B*(*E*_inc_) are obtained for 96 incident energies ranging from the pre-edge of the satellite at 7130 eV to a maximum of 10 000 eV. Since the *A*_*t*_ values from equation (4) are normalized, the *B*(*E*_inc_) values represent the intensity of the satellite. *B*(*E*_inc_) divided by the total *K*α spectrum intensity for a given incident energy results in the fraction of the total spectrum that is the *K*α_3,4_ satellite, *I*_sat_/*I*_*K*α_.

The *I*_sat_/*I*_*K*α_ ratio is the probability of an *n* = 2 two-body process occurring in the initial ionization of the atom. As this value increases, the validity of modelling the 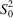 term as a constant in the XAFS equation, equation (1), decreases. The energy eigenvalues of the transitions involved cannot simply be modelled with a few Lorentzians, as often attempted empirically with many characteristic spectra. Fitting an *ab initio* spectrum ensures that all structure is captured in the satellite spectrum.

### Evolution of 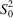


5.1.

The satellite evolution most commonly cited (Thomas, 1984 ▸) follows 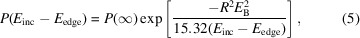
where *R* is the radius of the shell in ångstrom, all energies are in units of electronvolts, and the constant is given by 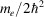 and scaled from metres to ångstrom.

Roy *et al.* (2001 ▸) developed a simple generic model of the evolution of the transition probabilities using the Slater form of one-electron wavefunctions of atomic orbitals and the sudden approximation, the ‘Roy’ model: 
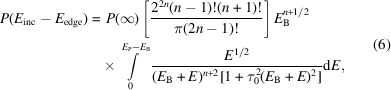
where *E*_inc_ is the energy of the incident photons, *E*_B_ is the binding energy of the shake-off orbital of interest, *n* is the principal quantum number of the shake-off shell, *E*_P_ is the energy of the photoelectron given by *E*_inc_ − *E*_edge_ and *P*(∞) is the shake-off probability in the high-energy limit, known from the results of the sudden approximation. τ_0_ is the characteristic time over which the interaction takes place and is also expressed as 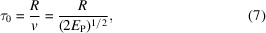
where *R* is the characteristic distance in the atom and represents the size of the orbital of the shake-off electron, with *v* being the velocity of the photoelectron. All variables in equations (6) and (7) are expressed in Hartree atomic units (*m*_*e*_, ℏ, *e*, 4πε_0_ = 1). There is a slight difference between equation (6) and its source, equation (16) by Roy *et al.* (2001 ▸). The original has a print error in the normalization, which is corrected here, as noted by Raboud *et al.* (2002 ▸).

Directly comparing models is difficult, equation (6) is based around a bound-free transition while the Thomas model is based around a bound–bound transition. Mukoyama *et al.* (2009 ▸) modified the Roy model for a bound–bound case where the electron is excited into an unoccupied bound state: 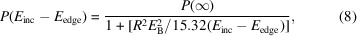
which is referred to as the Mukoyama model. The key difference in the derivation between equations (5) and (8) is in different definitions of the time dependence of the Hamiltonian.

When comparing such models to our experimental data, we leave both *P*(∞) and *R* as free parameters. Usually, when working with the Roy model, it is customary to take *R* to be the value of the maximum of the charge density of the Slater-type orbital, which would be 0.07417 Å. The value of the *K*-edge binding energy was taken to be 6539 eV (Bearden, 1967 ▸). *GRASP* returns a value of 6547.1 eV. For the binding energy of the 2*p* sub-shells we cannot take the standard values for Mn, as the 1*s* core hole will have significantly shifted the potential and, by extension, the binding energies of the remaining electrons. Previous comparisons and modelling of the binding energies of ionized atoms (Kawatsura *et al.*, 2003 ▸; Shigeoka *et al.*, 2004 ▸; Desclaux *et al.*, 1974 ▸) have suggested that the *Z* + 1 approximation provides good agreement with experiment. Therefore, we take the binding energies of the 2*p*_1/2_ and 2*p*_3/2_ sub-shells of Mn with a 1*s* hole to be equivalent to those of Fe (Fuggle & Mårtensson, 1980 ▸) minus 2% (Parratt, 1936 ▸), giving 692.7 and 705.5 eV, respectively. *GRASP* returns 706.53 and 721.05 eV. In this work, however, we do not model each of the 2*s*, 2*p*_1/2_ and 2*p*_3/2_ contributions individually, and so when choosing a value for *E*_B_ we simply take the value of the 2*p*_3/2_ shell, as it will be the most dominant. We also include an energy-offset term (Δ*E*) as a free parameter.

**Figure 15:**
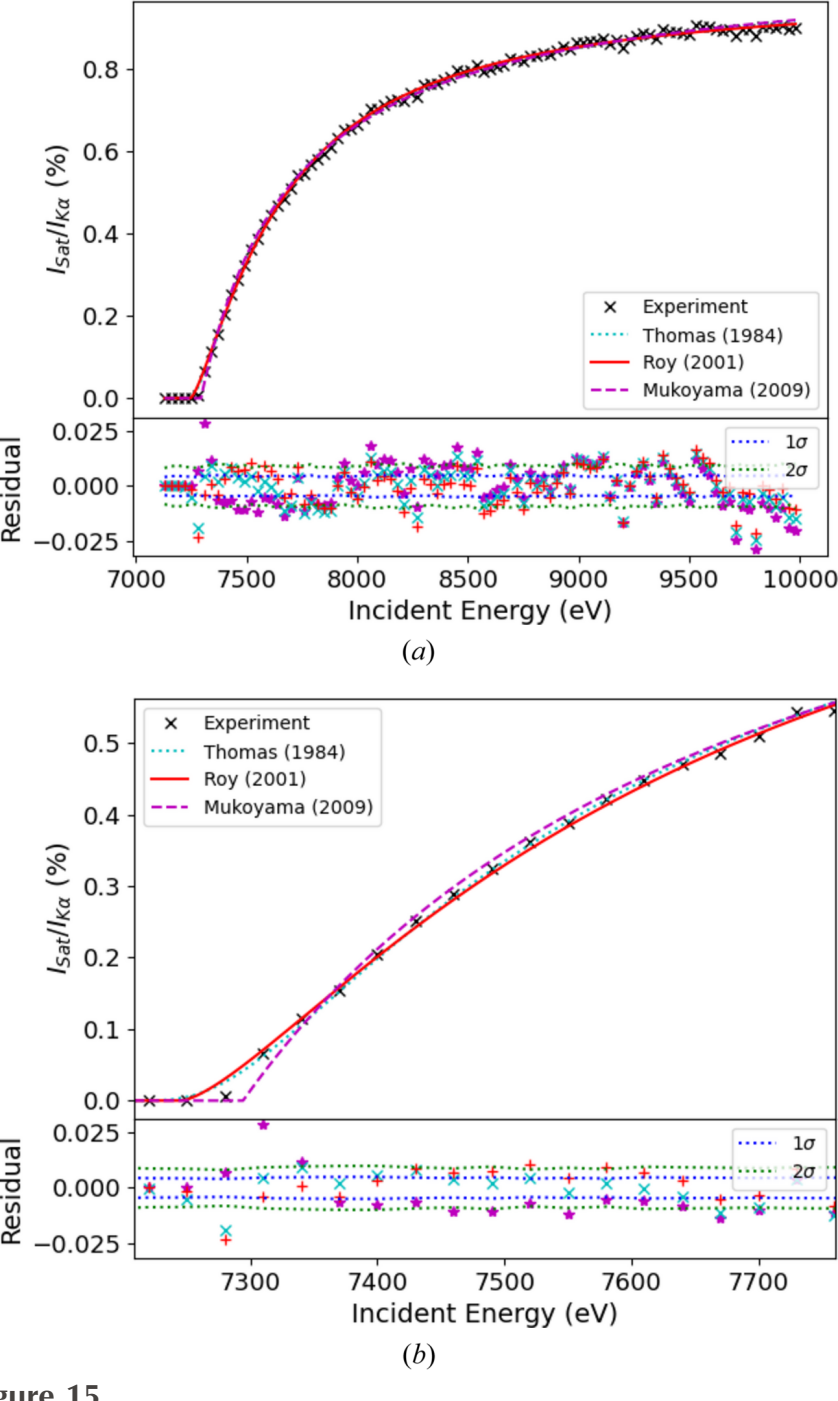
Comparisons of theoretical models with experimental data, plotted against the incident energy *E*_inc_. All the models show good agreement but the Roy model has the best fit with a 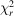 of 3.23.

Fits are shown in Fig. 15 ▸ and extracted parameters are shown in Table 1 ▸. The theoretical models are fitted to the experimentally derived values from fitting equation (4) to the XR-HERFD map. These fits resulted in the *I*_sat_/*I*_*K*α_ ratio.

### 5.2. Discussion and implications for future XR-HERFD experiments

All three evolution models are in good agreement with the data. The Roy model performs the best and the Mukoyama model performs the worst, particularly at low energies. All models return a value for the magnitude of the satellite as a percentage of the total emission spectra of between 1.02 and 1.07% in the high-energy limit, compared with 1.10% measured by Parratt (1936 ▸). Our *ab initio* combined 2*s* and 2*p* shake-off probability, as outlined in Section 3.1, was 1.108%, compared with 0.803% (Mukoyama & Taniguchi, 1987 ▸) and 1.43% (Kochur *et al.*, 2002 ▸) from the literature.

The Thomas and Mukoyama models both return similar values for the effective interaction radius (0.107 and 0.116 Å, respectively); however, the Roy model returns a value of roughly half that (0.0519 Å). These compare with a value of 0.0980 Å determined by performing *ab initio* calculations of the expectation value of the radius of the 2*p* orbital using *GRASP*. The similarity in results returned by the Mukoyama and Thomas models shows [as noted by Thomas (1984 ▸)] that the exact form of the time dependence of the Hamiltonian is not of critical importance provided 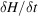 is near zero for all times except *t* = 0, where it is positive from some short time *t*_0_.

The fitted values of our 2*p*_3/2_ binding energy offset term Δ*E* give values of 643.06, 704.25 and 755.214 eV for the Thomas, Roy and Mukoyama models, respectively. This compares with *ab initio* calculations performed in *GRASP* of 721.05 eV.

While the Roy model returns the smallest 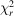 value of the three models investigated in this work, based on its significant discrepancy with the returned values from *GRASP*, we find that the fitted value for the radius is nonphysical. Thus we conclude that the Thomas model provides the best overall fit of the data, returning only a slightly higher overall 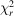 but returning much more plausible physical parameters, most notably with the radius.

A noteworthy feature in Fig. 14 ▸ is how the ratio values increase as incident energy increases. This makes sense as the 2*s* electron has a greater binding energy and therefore a later onset energy. The satellite intensities should emerge and grow towards a fixed value as the incident energy increases past the onset energy, which is observed in Fig. 14 ▸. Theoretical predictions of how the intensity evolves as a function of incident energy are an area for future work and empirical studies are the necessary path in the meantime. The evolution of the *K*α_3,4_ satellite as a hole is observed in several of the figures already presented in this work, but future work may be able to separate the individual components of the spectrum and observe their evolution, for the 2*s* and 2*p* shake-off satellites.

*K*α_3,4_ does not have the largest contribution to 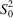, but is the most well resolved satellite from the main-diagram transitions and thus easiest to isolate and model. It does, however, demonstrate that other shake-off satellite transitions that are known to have much larger intensities and occur much closer to the absorption edge (6539 eV) will have a significant effect on 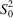.

The shake-off satellites that are unresolved from the diagram line include the set *nl* ∈ {3*s*, 3*p*, 3*d*, 4*s*}. Recent work on other 3*d* transition metals has shown that for scandium (Dean *et al.*, 2024 ▸) the intensity of these shake-off satellites amounts to 34.69% of the total *K*α_1,2_ spectrum and for copper (Nguyen *et al.*, 2022*b* ▸) the sum amounts to 25.51%. For manganese *K*α_1,2_, the two reported theoretically derived intensities of the non-resolved shake-off satellites are 32.03% from Kochur *et al.* (2002 ▸) and 25.86% from Mukoyama & Taniguchi (1987 ▸).

Each of these results have been calculated in the sudden limit where incident energy is large enough to cause an adiabatic electron loss, roughly two to three times the *K* edge. From the *K* edge, each shake-off satellite would have an onset energy equal to its binding energy; for example, the 3*p* shake-off satellite has an onset of 6539 eV (*K* edge) plus 47.2 eV (3*p* binding energy) (Fuggle & Mårtensson, 1980 ▸). The shake-off satellites evolve from zero intensity at the onset energy to the upper bound – as shown for *K*α_3,4_ in this work – providing an energy dependency to the many-body reduction factor that can be as large as 32.03% in manganese *K*α_1,2_ (Kochur *et al.*, 2002 ▸).

## 6. Conclusions

This work has presented XR-HERFD results for the Mn *K*α spectrum with a large range of incident energies and an extended emission axis in order to observe the *n* = 2 satellite in the high-energy tail of *K*α_1_. These results provide a clear pattern of the evolution of shake-off probabilities and in turn the energy dependence of the many-body reduction factor 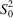. The origin of this *K*α_3,4_ spectrum has been debated in the literature, which is mostly due to the difficulty in performing necessary theoretical calculations to prove a specific spectral genesis. *Ab initio* calculations of [1*s*2*s*] → [2*p*2*s*] and [1*s*2*p*] → [2*p*]^2^ transitions in atomic manganese using the MCDHF method were performed, which resulted in eigenvalue spectra, Fig. 10 ▸. These eigenvalue spectra support the claim that the 2*s* and 2*p* shake-off events are at least contributing phenomena to the *K*α_3,4_ spectrum. To strengthen the claim that these shake-off events are the cause of the satellite, *ab initio* shake probabilities and Auger suppression factors have been calculated to reduce the need for free parameters. The results of the fitting are remarkable, with goodness-of-fit parameter 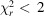 for almost all incident energies, with only two Lorentzian widths, one Gaussian broadening and a common scaling factor as free parameters.

This common scaling factor, *B*(*E*_inc_), has been defined for each incident energy, *E*_inc_, which has allowed for the evolution of the intensity of the *K*α_3,4_ profile relative to the full *K*α profile as a function of incident energy. The strong evidence that many-body quantum processes are the cause of the satellite spectrum suggests with equal strength that the many-body reduction factor, 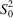, in the standard XAFS equation, equation (1), should be modelled as a function of incident energy, rather than as a constant.

We also presented one of the most rigorous tests of the existing theory of the evolution of satellite transitions, with comparisons to data accurate to 0.4692%, with over 90 points extending 2.3 keV beyond the onset energy of the satellite. This represents some of the most extensive and accurate measurements of satellite evolution to date. The high accuracy of our data enabled effective discrimination of the studied theories, with the Thomas (1984 ▸) model performing best overall with a 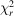 of 3.39 and returning values for the fitted parameters consistent with existing theoretical predictions. Furthermore, our extracted value for the high-energy shake-off probability using this model was found to be in agreement to within less than 6% of both previous measurements and our own *ab initio* calculations.

Our results demonstrate the extremely high accuracy that these models can achieve if the proper parameters are known accurately enough. With many measurements and calculations for the high-energy limits of satellite intensities already existing, we show it is possible to effectively model the contributions to the many-body reduction factor, 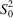, to spectroscopic analyses without the need for extensive measurements.

This work also, through modelling of the evolution of the *n* = 2 satellite, provides a method of probing and measuring the electron orbital. As the *n* = 2 orbital will not be significantly affected, this method could be of great use in observing and determining the effects of oxidation states on the internal electronic structure of molecules and compounds.

## Figures and Tables

**Figure 16:**
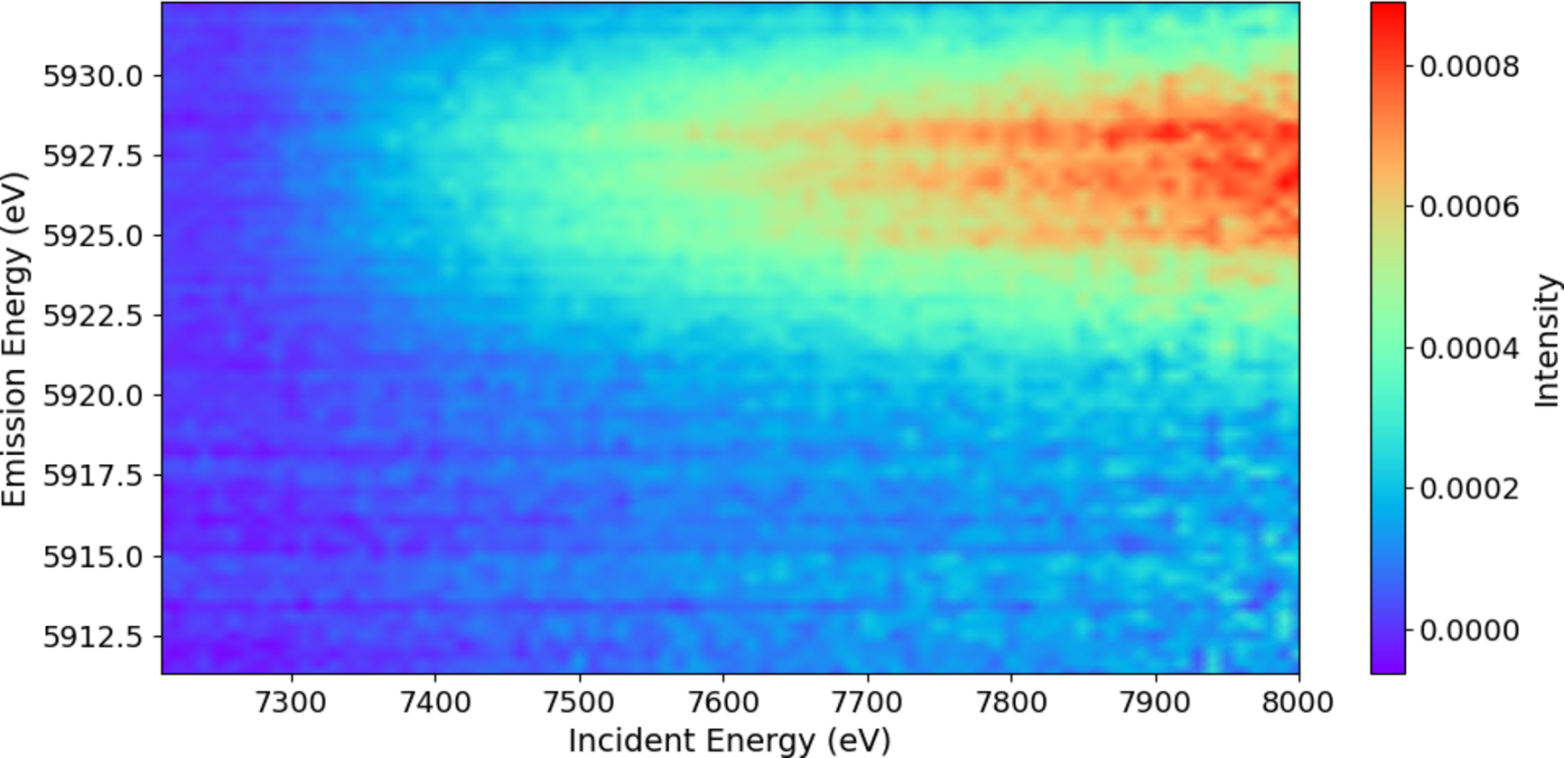
First observation of the satellite (Tran *et al.*, 2023 ▸), reprocessed following Sier *et al.* (2024 ▸), isolated from the Mn *K*α_1_ background and using the spliced data, showing the increase in intensity with energy above the onset.

**Figure 17:**
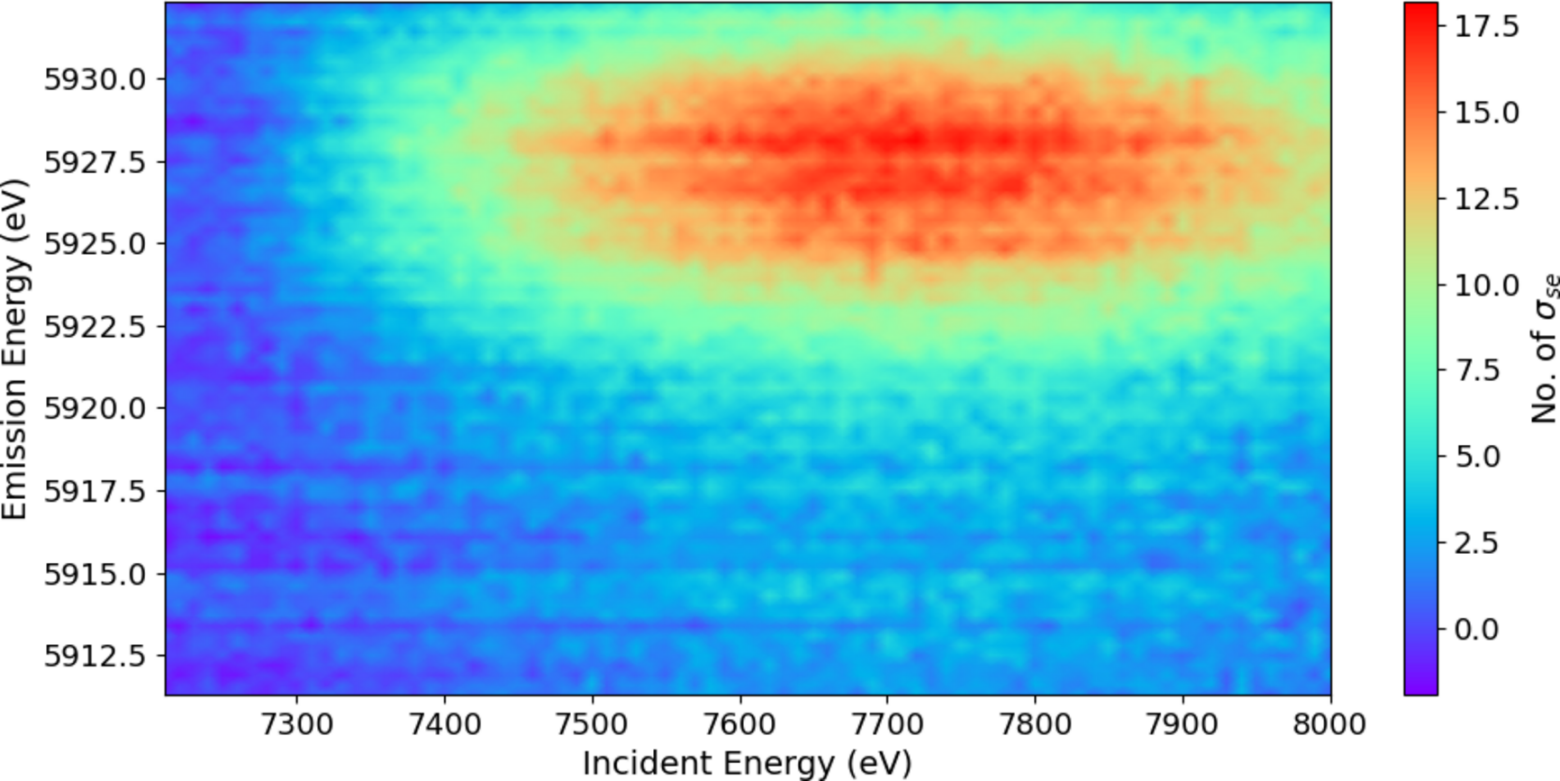
A significance plot of the signature of the satellite divided by the standard error uncertainty of the pooled data, using the spliced data, for the first observation of the satellite (Tran *et al.*, 2023 ▸), reprocessed following Sier *et al.* (2024 ▸).

**Figure 18:**
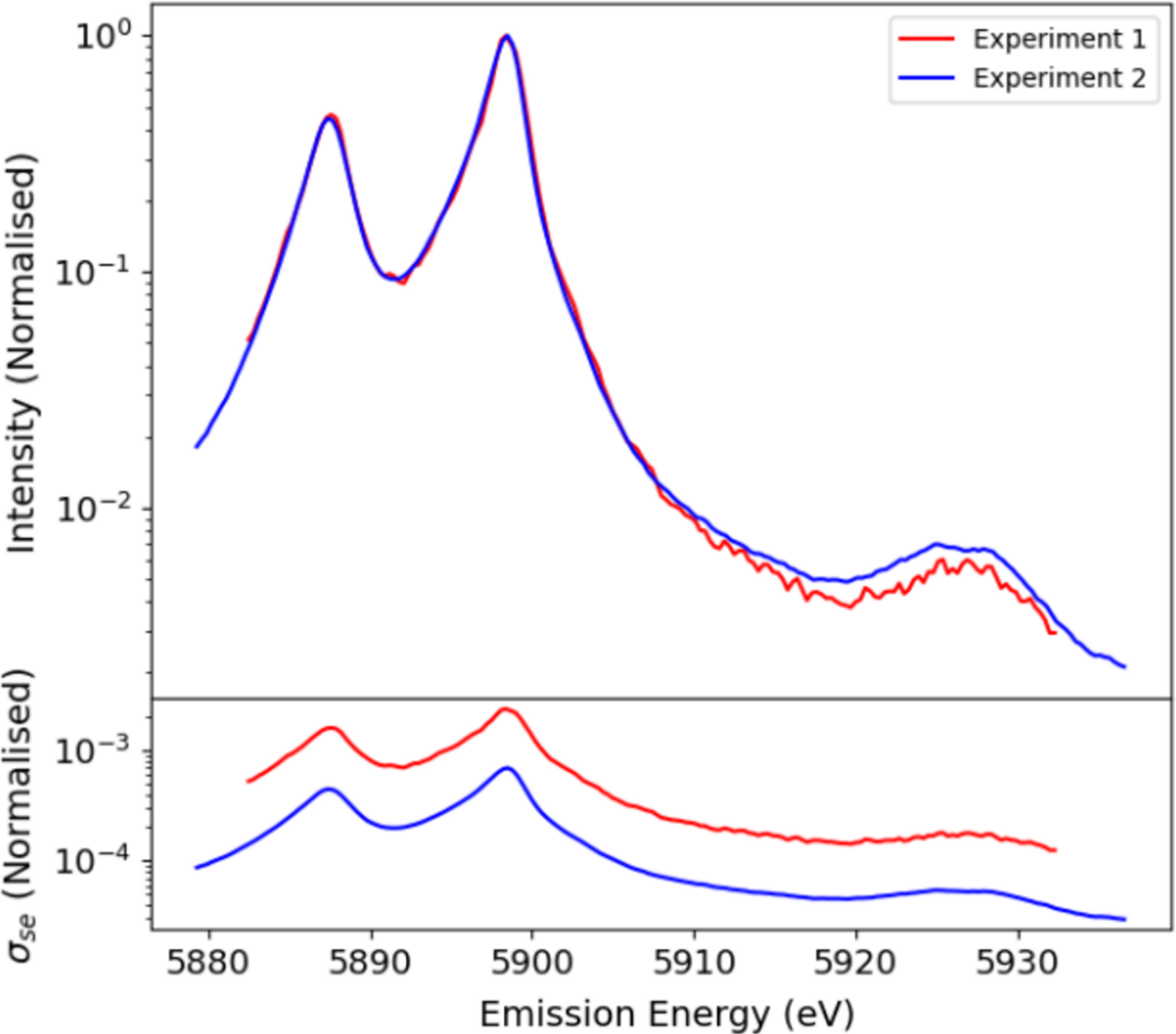
The original experimental spectrum versus *E*_em_ compared with the current measurement. Both have been spliced. The satellite has much greater clarity with greater statistics, and can clearly measure two constituent peaks, which is crucial for theoretical interpretation and measurement.

**Table 1:** Fitting parameters extracted from the Thomas, Roy and Mukoyama models for the evolution of the *n* = 2 satellite

Model	Thomas (1984 ▸)	Roy *et al.* (2001 ▸)	Mukoyama *et al.* (2009 ▸)
*P*(∞) (% of total)	1.041	1.026	1.066
*R* (Å)	0.1074	0.0519	0.1167
Δ*E* (eV)	49.60	−11.59	−62.55
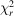	3.39	3.23	4.82

## Appendix A Comparison with earlier work

Tran *et al.* (2023 ▸) presents our first observation of this *n* = 2 satellite for Mn *K*α_1_ using the XR-HERFD technique. We can compare improved plots from that first observation using advanced techniques (Sier *et al.*, 2024 ▸). Even with the improved analysis, the earlier statistics, clearly a major discovery within RIXS, HERFD and XAFS investigations (Fig. 16 ▸), are much weaker and more limited than those in the advanced analysis (Fig. 17 ▸). A key experimental advance was to change the harmonic rejection mirror to avoid reducing the incident beam intensity significantly, improving statistics for the higher-energy spectra, and the expansion of the range to observe the signature and evolution much more clearly. Furthermore, the earlier statistics showed a satellite, mainly a broad single peak, and not much structure. Another significant advance was to splice the data to improve the resolution. The improved data, statistics and analysis clearly resolve and identify two peaks (Fig. 18 ▸). This is crucially important for the interpretation and understanding of theory and of the physical and chemical processes involved.
